# 2279. Appropriateness of Antibiotic Prescribing Before vs. After Minimum Inhibitory Concentration (MIC) Suppression in Culture Reports: A Quasi-experimental Study

**DOI:** 10.1093/ofid/ofad500.1901

**Published:** 2023-11-27

**Authors:** Huda Jamaan Aljedaani, Abrar K Thabit, Manea Al Munjem, Mohammed Bazuqamah

**Affiliations:** King Khlaid Hospital - Najran, Jeddah, Makkah, Saudi Arabia; King Abdulaziz University, Jeddah, Makkah, Saudi Arabia; King Khalid Hospital, Najran, Najran, Saudi Arabia; King Khalid Hospital, Najran, Najran, Saudi Arabia

## Abstract

**Background:**

Culture and susceptibility results are essential to identify the offending organism and optimize antibiotic treatment; hence, decrease the emergence of antibiotic resistance and achieve better clinical outcomes. Antibiotic prescribers should learn how to interpret MIC breakpoints to prescribe a suitable antibiotic as different antibiotics have different MIC breakpoints against different microorganisms. No study to date has evaluated the impact of MIC suppression in culture reports while keeping interpretation results on the appropriateness of antibiotics choice by non-infectious diseases prescribers. We assessed the impact of MIC suppression on the appropriateness of antibiotic prescriptions.

**Methods:**

This was a quasi-experimental study (pre-post intervention) that was conducted over 14 months on hospitalized patients in the medical floor who had a symptomatic infection with a positive culture and were prescribed a full course of definitive antibiotics by prescribers not specialized in infectious diseases pre and post MIC suppression in culture reports. The primary outcome was appropriateness of antibiotic selection (the de-escalation to the narrowest spectrum antibiotic to which the organism is susceptible with appropriate dose). Secondary outcomes were clinical cure (resolution of signs and symptoms, including defervescence and normalization of white blood cells count), length of stay (LOS), and antibiotics expenditure as defined daily dose (DDD) per 1,000 patient days and days of therapy (DOT) per 1,000 patient days.

**Results:**

100 patients were included, 50 in each phase. No difference was observed in the baseline characteristics, including age, sex, infection site, isolated organism, and prescribers’ departments. Appropriate antibiotic selection was significantly higher in the post-intervention phase compared to the pre-intervention phase (62% vs. 40%; *P*=0.028). LOS was shorter in the post-intervention phase compared to the pre-intervention phase (5 vs. 9.5 days; *P*=0.011). No difference was reported in the other outcomes.

**Conclusion:**

Our study showed that suppressing MIC values from cultures reports was helpful in choosing the appropriate antibiotic and minimizing the LOS. Future research is needed to confirm these findings.

**Disclosures:**

**All Authors**: No reported disclosures

